# Evaluation of Human Exposure to* Aedes* Bites in Rubber and Palm Cultivations Using an Immunoepidemiological Biomarker

**DOI:** 10.1155/2018/3572696

**Published:** 2018-08-09

**Authors:** Céline Mabot Yobo, Cécile Agnimou Malanfoua Sadia-Kacou, Maurice Akré Adja, Emmanuel Elanga-Ndille, André Barembaye Sagna, Négnorogo Guindo-Coulibaly, Anne Poinsignon, Franck Remoue, Benjamin Guibéhi Koudou

**Affiliations:** ^1^Institut Pierre Richet (IPR), Institut Nationale de la Santé Publique (INSP), 01 BP 1500, Bouaké 01, Côte d'Ivoire; ^2^Université Nangui Abrogoua, 02 BP 801, Abidjan 02, Côte d'Ivoire; ^3^Laboratoire de Zoologie et de Biologie Animale, UFR Biosciences, Université Félix Houphouët Boigny, 22 BP 582, Abidjan 22, Côte d'Ivoire; ^4^Institut de Recherche pour le Développement (IRD), Maladies Infectieuses et Vecteurs: Ecologie, Génétique, Evolution et Contrôle (MIVEGEC), UMR IRD 224-CNRS.5290, University of Montpellier, Montpellier, France; ^5^Liverpool School of Tropical Medicine, Pembroke place, Liverpool L3 5QA, UK

## Abstract

Arbovirus infections, mainly transmitted by* Aedes *mosquito, are emerging in Africa. Efficient vector control requires an understanding of ecological factors which could impact on the risk of transmission, such as environmental changes linked to agricultural practices. The present study aims to assess the level of human exposure to* Aedes *mosquito bites in different agroecosystem area, using an immunological tool which quantifies human IgG antibody response to one* Ae. aegypti* salivary peptide. Specific IgG responses were assessed during dry and rainy seasons, in children living in different villages in Côte d'Ivoire: N'Zikro (rubber and oil palm exploitations), Ehania-V5 (oil palm), and Ayébo (without intensive agricultural activities). In the dry season, specific IgG levels were significantly lower in Ayébo compared to Ehania-V5 and N'Zikro and, interestingly, were similarly high in both villages with cultivations. In the rainy season, no difference of specific IgG was observed between villages. Specific IgG responses remained therefore high during both seasons in villages associated with intensive agricultural. The rubber and oil palm cultivations could maintain a high level of human exposure to* Aedes *mosquito bites during both dry and rainy seasons. These agricultural activities could represent a permanent risk factor of the transmission of arboviruses.

## 1. Introduction

*Aedes *mosquitoes, such as* Aedes aegypti* and* Ae. albopictus,* are vectors of the major arboviral infections including yellow fever, dengue, chikungunya, and Zika. These diseases represent important threats to human health worldwide [1]. Despite the availability of an effective vaccine against yellow fever, cases of outbreaks have been reported regularly since 2004, in several African and south American countries [2]. Similarly, outbreaks of dengue and chikungunya infections have significantly increased in Africa, sometimes, in the severe forms [1, 3, 4]. Moreover, the recent Zika epidemic in the world highlights the impact of arboviruses on public health and their specific risks in Africa, where fever is often wrongly associated with malaria infection.

In Côte d'Ivoire, several cases of dengue and yellow fevers were reported in various parts of the country, during the past two decades [5–9], and* Ae. aegypti* mosquitoes were found to be the main vector [5, 8]. Some studies also highlighted the increased circulation of yellow fever virus and several dengue serotypes in human populations and in the* Aedes* mosquitoes [5]. In addition, the presence of* Ae. albopictus* has been reported in Abidjan by recent studies [10, 11]. These data highlighted that the risks of dengue and yellow fever epidemic remained high in this country, as recently observed with dengue epidemic in Abidjan in 2017.

The transmission of arboviruses is strongly dependent on environmental factors. Any changes to the natural environment through urbanization, hydrological, or agricultural changes could result in a variation in the diversity of vectors, their densities, and their spatial distribution [12]. Several studies have shown that some agricultural practices (banana, taro, and pineapple) can favor the larval development of* Aedes* mosquitoes. Some crops with leaf sheathes can create cavities capable of maintaining stagnant water, allowing the development of mosquito larvae. Previous studies showed thus the presence of larvae of several* Aedes* vector species including* Ae. aegypti* and* Ae. albopictus* in latex harvesting pots [13, 14]. All these results confirm the amplifying impact of certain crops on the population of* Aedes* including some vectors and from this, the potential increase of arbovirus transmission risk in cultivated areas.

Currently, with the exception of yellow fever which could be controlled by adequate vaccine coverage, no vaccine and specific therapeutic drugs are available to treat other arboviral infections. Therefore, vector control remains the only method for reducing or preventing transmission. In endemic areas, a major challenge is to make a link between environmental modification and the transmission risk of these diseases. For Côte d'Ivoire, a country dependent largely on agriculture, this link has not been established, especially in the southeast part of the country where large oil palm and rubber farms have been created since several decades. However, the previous entomological investigations in one area of Aboisso region revealed the presence of several species of* Aedes*, including* Ae. aegypti*, the main vector of human arboviruses, with two others potentials vectors:* Ae. africanus* and* Ae. vittatus *[15].

Assessing the risk of arbovirus transmission is traditionally based on entomological methods. These techniques focus on the identification of breeding sites and/or the collection of adult mosquitoes, to indirectly estimate the human-vector contact. Unfortunately, these methods present several limitations which make extrapolation to the actual estimation of human exposure to bites of* Aedes* mosquitoes [16] and the evaluation of human-vector contact at the individual level impossible.

To overcome these limitations, new sensitive and complementary tools to evaluate the individual human exposure to* Aedes* bites have been developed during last decade. Numerous studies have demonstrated that the human antibody (Ab) response to blood-sucking arthropod salivary proteins could represent a pertinent approach for assessing the human-vector contact. Indeed, human Ab responses against whole saliva have been described as a suitable biomarker of human exposure to the bites of many vectors such as, ticks [17] sand flies [18, 19],* Glossina *[20], and mosquitoes [21–23]. Even though this approach may appear to be valid, whole saliva of arthropod vector could not be used as pertinent antigens because of many factors: (i) a potential cross-reactivity with other hematophagous arthropods, (ii) a lack of reproducibility between saliva batches, and (iii) the inadequate production needed for large scale studies. In the objective to optimize this promising indicator, the identification of specific and antigenic salivary proteins and/or peptides has been done. With respect to* Aedes *species, the putative 34 kDa family secreted salivary protein appeared to be antigenic and specific to* Aedes *genus [24]. Interestingly, one peptide (Nterm-34 kDa) from this protein of* Ae. aegypti *saliva was recently identified (*Ae. aegypti *Nterm-34 kDa peptide) and validated as a pertinent and specific candidate biomarker of human exposure to bites of major* Aedes *species (*Ae. aegypti*,* Ae. albopictus*) through several studies implemented in different epidemiological and entomological contexts [25–27].

The present study aimed to evaluate human exposure to* Aedes* bites in different agroecosystem such as oil palm and rubber plantations in the health department of Aboisso, in southeast Côte d'Ivoire, using this salivary immunoepidemiological biomarker. Specifically, it was used (i) to compare human-*Aedes* contact from intensive farming villages to that of the village where there are no intensive agricultural activities and (ii) to evaluate the evolution of this exposure level between the dry and the rainy seasons, corresponding traditionally to a low and high exposure seasons to* Aedes* bites, respectively.

## 2. Materials and Methods

### 2.1. Ethics Statement

This study followed ethical principles recommended by the Edinburgh revision of the Helsinki Declaration. The Director of Health District of Aboisso administrative Department and authorities of each studied village were informed about objectives, procedures, and benefit of the study. Approval, including the use of oral consent, was obtained from the different village authorities and the Departmental Director of Health of Aboisso district before starting data collection. Because of initially being integrated in malaria survey, this study was approved by the National Malaria Control Program (NMCP) of Côte d'Ivoire. In addition, the participation of children in the study including the blood sample collection was done after the oral informed consent of each parent or guardian of children. The data generated during the study were treated confidentially. Sick children were treated according to the National Policy. This study did not involve endangered or protected species.

### 2.2. Study Area

**Figure 1:**
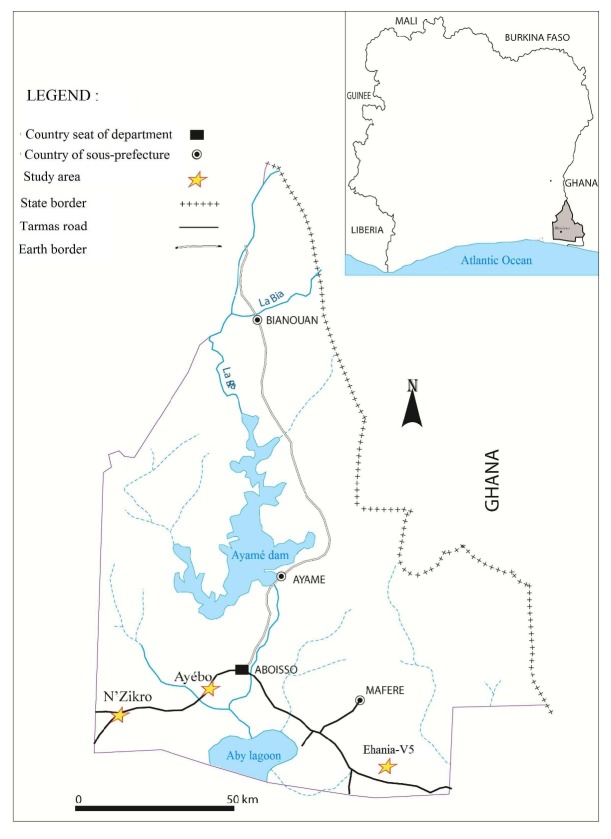
Map of study areas in Aboisso department located in southeastern Côte d'Ivoire. The immunological study was conducted in three different agroecosystem villages represented by stars. N'Zikro is a large farming area including oil palm and rubber (Hevea brasiliensis) plantations. Ehania-V5 (V5) is located in the center of the oil palm plantation (Elaeis guineensis). Ayébo village located far from large farms represented the control site of the present study (distant to cultivation activities).

This study was conducted in the Aboisso health district, in southeastern of Côte d'Ivoire (Figure 1), located at 122 km from Abidjan (05°28′ north latitude and 03°12′ west longitude). This region is characterized by an equatorial climate supported by abundant rainfall. The average annual precipitation is 1848 mm with an annual temperature around 27°C. The climate is punctuated by two major seasons: the long rainy season extends from April to July, with the peak rainfall in June, while the long dry season extends from October to March.

Three villages of Aboisso district, characterized by different agroecosystems, were selected for the study: N'Zikro, Ayébo, and Ehania-V5. Ehania-V5 belongs to the Ehania's Integrated Agricultural Unit: IAU of the PALMCI of Côte d'Ivoire. According to the Livret-Palmci report of 2011, the IAU of Ehania comprises 11,544 ha of palm fields [28]. Ehania-V5 (V5) is located in the center of the oil palm plantation (*Elaeis guineensis*). N'Zikro (59.4 km from Ehania-V5) is a large farming area including oil palm and rubber (*Hevea brasiliensis*) plantations. The plantations are located near the village. Large traditional rubber fields that border the village in the southeast, east and north parts extend to about 200 ha. On the south-western side of the village are the PALMCI, industrial plantations of Toumanguié, which cover 3,600 ha of palm trees. Ayébo village located far from large farms represented the control site of the present study (distant to cultivation activities and to other studied villages: 18.5 Km from N'Zikro and 40.8 Km from Ehania-V5). The agricultural activities encountered within 800 meters of this village are limited to some plots of cassava, vegetable, yam, and corn. Ayébo could be considered as “control site” of the present study without intensive agricultural activities.

### 2.3. Study Design and Studied Population

Two cross-sectional surveys were conducted in November 2013 (dry season) and in July 2014 (rainy season) in the four study villages. As the present study was integrated to a malaria epidemiological survey, the studied population consisted of children aged from 6 months to 14 years living in these villages during the last two months before each survey. This time period was considered in the objective to exclude the study of the children who could present Ab response to* Aedes* salivary peptide from their previous place of residence. As integrated to malaria survey, one of the inclusion criteria was that selected children should not present any clinical sign of severe malaria according to WHO definition [29] and of any other disease that can cause an obvious febrile illness. Moreover, standardized questionnaires were individually administrated for assessing epidemiological information such as use of individual protection against mosquito bites, sex, and age. During the first survey in the dry season, 100 children were surveyed in N'Zikro and Ayébo, whereas only 80 were recruited in Ehania-V5. In the rainy season, the survey covered 102 children in N'Zikro, 99 children in Ehania-V5, and 100 children in Ayébo. The children surveyed in the rainy and dry seasons were not the same individuals. During each visit, a dried blood spot (DBS) was collected from each individual (on Whatman 3MM filter paper) for immunological analysis. All filter papers were kept at 4°C before being used.

### 2.4. Salivary Peptide Nterm -34 kDa

The Nterm-34 kDa salivary peptide from* Ae. aegypti* was selected as previously described [25]. The Nterm-34 kDa peptide used in this study was synthesized (purification > 95%) by Genepep SA (St-Jean de Vedas, France). It was shipped in lyophilized form and then resuspended in milliQ water and stored in aliquots at −20°C until their use.

### 2.5. Evaluation of Human IgG Antibody Levels (ELISA)

Enzyme-linked immunosorbent assays (ELISA) were performed on DBS eluates as previously described [25]. All ELISA conditions were determined after several preliminary experiments to obtain optimal dilutions of all reagents. DBS were first eluted by incubation in 350 *μ*l of Phosphate Buffer Saline (PBS) + Tween 0.1% (Sigma Aldrich, St. Louis, MO) at +4°C for 24 h. The peptide (20 *μ*g/ml in 100 *μ*l of PBS) was coated for 2.5 h at 37°C onto Maxisorp plates (Nunc, Roskilde, Denmark). Plates were blocked with 300 *μ*l/well of Protein-Free Blocking-Buffer (Pierce, Thermo Scientific, France). Each eluate was incubated in duplicate at 4°C overnight at a 1/20 dilution in PBS-Tween 1%. Mouse biotinylated Ab to human IgG (BD Biosciences, San Diego, CA) was incubated at a 1/2000 dilution in PBS-Tween 1% and peroxidase-conjugated streptavidin (GE Healthcare, Orsay, France) was added at a 1/2000 dilution in PBS-Tween 1%). Colorimetric development was carried out using 2,2′-azino-bis (3-ethylbenzthiazoline 6-sulfonic acid) diammonium (ABTS; Thermo Scientific, France) which is a substrate of peroxidase added to hydrogen peroxide (H_2_O_2_). Absorbance Optical Density (OD) was measured at 405 nm. In parallel, each tested sample was assessed in a blank well containing no salivary peptide antigen (ODn) to measure nonspecific reactions. A known positive control was included on each ELISA plate to control the plate-to-plate variation as well as reproducibility of the test. Individual results were expressed as the ΔOD value calculated according to the formula: ΔOD = ODx − ODn, where ODx represented the mean of individual OD values in the two wells containing antigen and ODn the OD value in well without antigen.

### 2.6. Statistical Analysis of Data

All data were analyzed with GraphPad Prism5 software (San Diego, CA). After ensuring that ΔOD values were not normally distributed, the nonparametric tests were used to compare ΔOD between groups. Mann–Whitney test was used for the comparison of Ab levels between two independent groups and the Kruskal-Wallis test was used for the comparisons between more than two independent groups. The Dunn's test was used for multiple pairwise comparisons between villages. All differences were considered significant at P <0.05.

## 3. Results

### 3.1. Characteristics of the Study Population

Altogether, 280 children were selected in dry season (the first survey) and 301 in rainy season. The age means ([95% CI]) were similar between the 3 studied villages in dry season (5.4 [4.6-6.1] in N'Zikro; 5.8 [5-6.6] in Ehania-V5; 7.3 [6.6-8.1] in Ayébo) and in rainy season (6.3 [5.7-7] in N'Zikro; 5.9 [5.1-6.6] in Ehania-V5; 7.1 [6.3-7.9] in Ayébo). The male/female sex ratios in the dry season were 0.89; 0.67; and 1.04 in N'Zikro, Ehania-V5, and Ayébo, respectively. In the rainy season, the sex ratios observed were 0.73; 1.15; and 0.67 in these same villages.

### 3.2. IgG Response to Nterm-34 kDa Salivary Peptide according to Villages in the Dry Season

**Figure 2:**
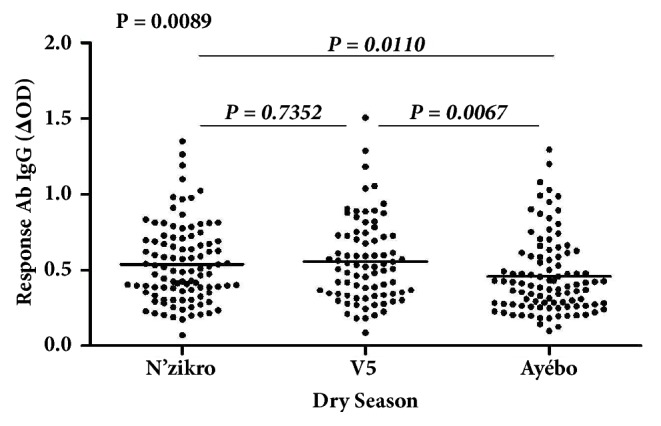
IgG Ab level to Nterm-34 kDa salivary peptide in individuals according to villages in dry season. Individual specific IgG (ΔOD) are represented in black points and the bars indicate the median values. The Kruskal-Wallis test was used to compare the level of specific IgG between the three villages.

Specific IgG responses to Nterm-34 kDa peptide were evaluated and compared according to the three villages during the dry season (Figure 2).

Results showed considerable variations within and between study sites. Despite the high interindividual heterogeneity observed in each village, specific IgG level was more pronounced in villages with intensive agricultural practice compared to the Ayébo “control site” without intensive agricultural activities. Statistical analysis showed that the medians of specific IgG Ab levels were significantly different according to these villages (P=0.0089; Kruskal-Wallis test). No significant difference was observed in the level of IgG Ab response between N'Zikro and Ehania-V5 which represented the “cultivated villages” but the specific IgG response in Ayébo appeared slightly lower compared to the other study villages (median Ayébo = 0.4020; median Ehania-V5 = 0.5250; median N'Zikro = 0.4950). Statistical analysis (Mann–Whitney test) confirmed that the IgG Ab level in Ayébo was significantly lower than those recorded in Ehania-V5 (P = 0.0067, Mann–Whitney test) and N'Zikro (P = 0.0110, Mann–Whitney test). In contrast, the specific IgG response observed in these last two villages was statically similar (P = 0.7352).

### 3.3. Evolution of IgG Ab Response to Nterm-34 kDa according to Villages in the Rainy Season

**Figure 3:**
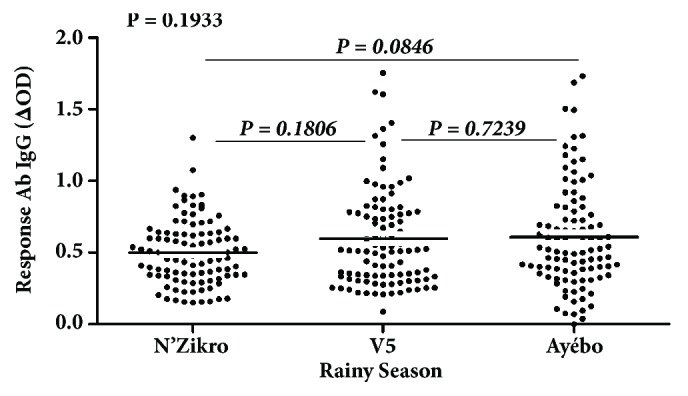
IgG Ab level to Nterm-34 kDa salivary peptide in individual according villages in rainy season. Individual specific IgG level (ΔOD) evolution has been evaluated in rainy season according to the three studied villages. The Kruskal-Wallis test was used to compare the level of specific IgG between these villages.

The specific IgG levels were also evaluated and compared according to villages during the rainy season. The medians of specific IgG Ab levels fluctuated from 0.4870 (in N'Zikro) and 0.5140 (in V5) to 0.5250 (in Ayébo). The statistical analysis showed no significant difference between these specific IgG Ab levels (P = 0.1933; Kruskal-Wallis test). Mann–Whitney test was also carried out to compare all pair of villages. The result showed any significant difference of the specific IgG responses between villages (Figure 3). Contrary to the result observed in the dry season, all these results suggest that the specifics IgG Ab levels were similar between the three villages during rainy season. They remained high and similar between the intensive agricultural villages as observed previously in the dry season (P = 0.1806, Mann–Whitney test). In contrast, the IgG Ab levels between the control village and the two intensive agricultural villages presented different evolution in this season compared to that observed in dry season. IgG Ab levels evaluated in the control site during the rainy season appeared similar to those of the intensive agricultural villages (Figure 3).

### 3.4. Evolution of Specific IgG Response in Each Village according to the Season

**Figure 4:**
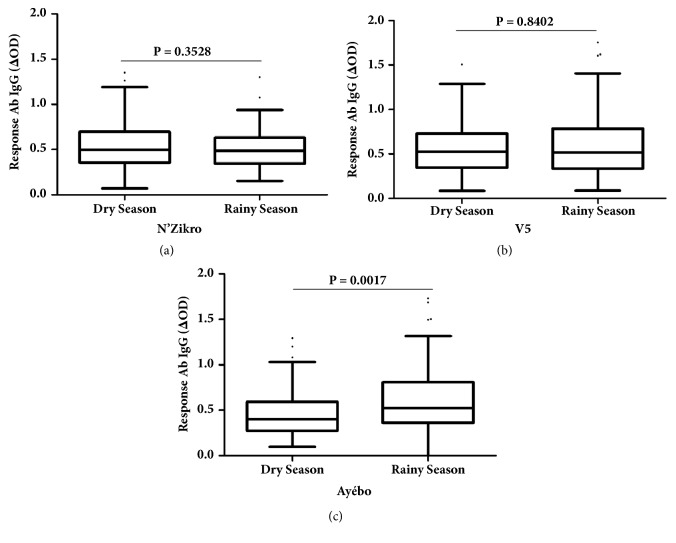
Evolution of IgG Ab response to Nterm-34 kDa salivary peptide between dry and rainy season in the villages. The evolution of specific IgG levels was evaluated in the population according to the seasons and presented in each village: N'Zikro** (a)**, V5** (b),** and Ayébo** (c). **Statistical comparisons of IgG levels between the dry season and the rainy season in each village were made by the nonparametric Mann–Whitney test.

The evolution of the IgG response to* Aedes* salivary peptide was then evaluated between dry and rainy seasons in each village. Overall, no difference in the level of specific IgG Ab was observed between the dry and the rainy seasons when studied participants from the three villages were merged (P = 0.0962; Kruskal-Wallis test, data not shown). When the comparison was done within each village, the median of specific IgG level in villages with intensive agricultural practices fluctuated from 0.4950 in dry season to 0.4870 in rainy season in N'Zikro (Figure 4(a)), and from 0.5250 to 0.5140 in Ehania-V5 (Figure 4(b)), respectively. The statistical analysis showed that the specific IgG responses did not significantly vary between dry and rainy seasons in these two villages. The specific IgG Ab levels of study populations in these intensive agricultural villages appeared thus similar in both seasons. By contrast, in Ayébo “control site”, the specific IgG levels significantly increased (Figure 4(c)) in the rainy season (median = 0.5250) compared to the dry season (median = 0.4020) (P= 0.0017, Mann–Whitney test).

Overall, these results indicate that specific IgG responses remained at high and similar levels during both dry and rainy seasons in agricultural villages. In contrast, in the control site, without intensive agricultural practices (Ayébo village), the specific IgG response showed significant seasonal variations. It increased from a lower level during the dry season to a higher level in the rainy season. The use of the studied personal protection against mosquito (mainly Insecticide Treated Net) did not influence the evolution of specific IgG level according to villages and season (data not shown). In addition, the specific IgG levels were evaluated according to two different age groups in studied children: (i) youngest age target (0-4 years) which potentially benefit from maternal protection against mosquito bites and which not move from their households and (ii) older children (4-14 years), which could move from their households within the village (i.e., to go to school for example) in the early morning and/or in the nightfall, the hours of major* Aedes* exposure. No differences in the evolution of specific IgG level according to the seasons were observed between both age groups whatever the studied villages (data not shown). Indeed, the same pattern of whole population (i.e., no evolution of IgG level in agricultural villages and increase of IgG level in Ayébo “control site” in rainy season compared to dry season) was observed in both age groups.

## 4. Discussion

Several studies have reported the influence of agricultural practices, such as irrigated rice cultivation [30] and market gardens [31], on the ecology of malaria vectors and their impact on the transmission of the disease. In addition, other studies have revealed effect of certain crops on the reproduction of mosquito vector species of arboviruses [14, 32, 33]. In the present study, we have highlighted the potential impact of the cultivation of palm and rubber on human exposure to* Aedes* mosquitoes bites, by using an immunoepidemiological biomarker assessing the Human-*Aedes* contact. This tool is based on the measurement of human IgG Ab response to the* Aedes *Nterm-34 kDa salivary peptide in children living in the studied area. This salivary peptide is highly conserved between studied* Aedes* species [25–27] and* Ae. aegypti* was the major anthropophilic* Aedes *species in the studied area and no* Ae. albopictus* was here identified ([34] and personal communication). Nevertheless, we cannot exclude that the observed specific IgG response could evaluate the exposure to other* Aedes* species and could be thus underestimated. Future relevant entomological investigations could be performed in the studied areas to highlight the present results.

Immunological analysis showed that the evolution of children IgG Ab response to* Aedes* bites was different between the control site and the intensive agricultural ones during the two study periods. In the dry season, the specific IgG Ab response was high and similar between the two villages where agricultural activities were intense, whereas the children living in Ayébo village, where agriculture is not intensively practiced, presented lower IgG Ab level. These observations suggest that although children living in the three study sites were exposed to* Aedes* bites, during dry season, the level of exposure was higher in the two large agricultural practiced sites than in the control site. However, in the present study, a different impact of rubber versus palm cultivation could not be highlighted because the absence of studied village presenting only rubber plantation. Future investigations will have to be carried out to highlight the specific impact of this or that plantation and will be performed in other study areas to confirm our hypothesis.

Interestingly, the IgG response to salivary antigen remained similarly high in the rainy season and dry seasons in the two studied intensive agricultural villages. In contrast, the Ayébo control village presented a significant increase of specific IgG Ab levels in the rainy season compared to the dry season. These observations suggest that Human-*Aedes* exposure was influenced by rainfall only in the control village. It could be here noticed that rainfall intensity was closely similar between the 3 studied villages because of their geographical proximity. It was low in dry season and became intense in rainy season. Similar observations were reported in previous studies carried out in a rural area of Benin. During these studies, the high level of specific IgG response was observed only in rainy seasons [25]. By consequences, the increase of human exposure to* Aedes* bite level in our control site may be induced by an increase of the* Aedes* mosquito density classically observed during the rainy season. Variations of the density of mosquitoes depend on several factors including rainfall patterns [35]. For* Aedes* mosquitoes, repeated rains result in the filling of open containers (domestic, peridomestic, or natural containers) and cavities capable of retaining water [36] which are suitable for their larval development. This same observation was made by Guindo-Coulibaly et al. [34] in Côte d'Ivoire, who reported that the abundance peak of* Aedes* eggs in the rainy season is a result of the proliferation of its breeding sites.

Interestingly, the impact of rainy season on* Aedes *exposure was not observed in the two villages with intensive agricultural practices. It suggested that these areas, with rubber or oil palm plantations, did not represent the classic situation/pattern of exposure to* Aedes *bites. On the contrary, the IgG Ab level remained high through the year in individuals living in these specific agricultural areas. Therefore, rubber and palm oil plantations could maintain high levels of exposure to* Aedes* bites among children living in these areas, as indicated by the original use of the specific salivary biomarker. We cannot exclude that other environmental or epidemiological factors could influence the immunological results. For example, one bias of the present analysis is that the studied population was not the same (same individuals) in dry and rainy season, even if it was children living in the same village. Nevertheless, the assessment of specific IgG response represents a “picture”, a proxy of human exposure in each village at the population level in each season.

The cultivation of rubber and palm can offer adequate conditions for the development of* Aedes *larvae. Indeed, several studies carried out in rubber plantations have demonstrated the effect of this type of cultivation in increasing the density of* Aedes*. Paily et al., working in a rubber plantation area in India, have highlighted the presence of* Ae. albopictus *larvae in the holes of rubber wood [37]. The rubber latex harvesting pots are also favorable breeding sites for* Aedes* mosquitoes. A recent work carried out in experimental rubber plantations of Anguédédou in Côte d'Ivoire reported the presence of larvae of* Ae. opok* mosquitoes in these pots [8]. Other studies have shown the presence of* Ae. aegypti* [13] and* Ae. albopictus* [14, 33] in the rubber harvesting latex pots.

The latex harvesting pots could collect rainwater. In Aboisso department, where the rains did not fail even if it was few in dry season, rainwater can often be found in latex pots. This water retention was not automatically emptied according to the routine activities and therefore can be used as breeding sites by several species of mosquitoes, especially* Aedes* species. With respect to the oil palm plantations, the axils of fresh leaves could be potential harborages for the larval development of these mosquitoes. The high level of child exposure to* Aedes* bites in intensive cultivation villages during both seasons can be then explained by the regular abundance of these mosquito species due to the proliferation and persistence of breeding sites in these particular cultivated ecosystems. This could therefore induce a permanent high risk of transmission of arbovirus infections for individuals living and/or working in the intensive cultivation of rubber and palm oil. The population, mostly living nearby the field or in dormitory villages located in the center of plantation, could represent the most vulnerable population at risk of transmission.

## 5. Conclusion

In Aboisso district, children are exposed to* Aedes* bites including* Ae. aegypti*, the main vector of dengue and yellow fever in Côte d'Ivoire. The present study suggests that the rubber and oil palm plantations could maintain the high level of exposure to* Aedes* bites during the dry and the rainy seasons. These agricultural practices could, therefore, represent a permanent risk of arbovirus transmission. The maintenance of the high level of* Aedes *exposure in these areas could be favored by several factors: the type of crop, its extent, and practiced agricultural behavior.

## Data Availability

The data used to support the findings of this study are available from the corresponding author upon request.

## Abbreviations

IAU: Integrated Agricultural Unit
PALMCI: Côte d'Ivoire palm oil industry
ELISA: Enzyme-linked immunosorbent assay
OD: Optic density
DBS: Dry blood spot.
